# Healthcare providers' preconception care practice and associated factors in Ethiopia: a systematic review and meta-analysis

**DOI:** 10.3389/frhs.2025.1226206

**Published:** 2025-05-15

**Authors:** Agerie Mengistie Zeleke, Worku Chekol Tassew, Getaw Wubie Assefa, Yeshiwas Ayale Ferede

**Affiliations:** ^1^ Department of Midwifery, Debark University College of Health Science, Debark Town, Ethiopia; ^2^ Department of Nurse, Tedda Health Science College, Gondar, Ethiopia; ^3^ Department of Midwifery, Tedda Health Science College, Gondar, Ethiopia

**Keywords:** practice, preconception care, healthcare, providers, Ethiopia

## Abstract

**Introduction:**

Providing preconception care through good practice by healthcare providers is very important in reducing maternal and child mortality and morbidity. However, there are no available detailed review articles in Ethiopia. Therefore, the purpose of this study was to ascertain the level of knowledge of preconception care and related factors among Ethiopian healthcare providers.

**Methods:**

A variety of data sources, such as PubMed, Scopus, African Journal Online (AJOL), Google Scholar, and Semantic Scholar, were used to identify studies published until March 2023. This review was performed in accordance with the Preferred Reporting Items for Systematic Reviews and Meta-Analyses (PRISMA) guidelines. The data were subsequently transferred to STATA software version 11 for further data analysis. A random-effects model was used to estimate the prevalence of practice about preconception care, and the results are reported in a forest plot.

**Results:**

Eight studies involving 3,848 healthcare providers were included. The pooled prevalence of a good level of practice of preconception care among healthcare providers in Ethiopia was 53.54 (95% CI: 45.09, 61.98), *p* < 0.0001. Healthcare providers who had a degree and above educational status (AOR = 4.83; 95% CI: 1.80, 12.96), those working at hospital health facilities (AOR = 2.97; 95% CI: 2.07, 4.27), those ever reading preconception care guidelines (AOR = 3.49; 95% CI: 2.39, 5.07), availability of libraries at health facilities (AOR = 5.59; 95% CI: 2.84,11.04), trained healthcare providers on HIV counseling and testing (AOR = 6.86; 95% CI: 3.75, 12.53), and trained healthcare providers on preconception care (AOR = 6.19; 95% CI: 4.23, 9.06) were predictors of a good level of practice with preconception care.

**Conclusions:**

Nearly half of healthcare providers do not have good practice of preconception care in Ethiopia. Therefore, healthcare providers' knowledge of preconception care should be improved through preconception care, HIV counseling and testing training, and access to guidelines, libraries, and the internet at nearby health facilities, especially at health centers. Finally, stakeholders collaborate with policymakers to develop strategies to improve healthcare providers' preconception care practice.

## Introduction

According to the World Health Organization (WHO), preconception care (PCC) comprises a range of interventions aimed at identifying and modifying medical, behavioral, and social health risks among individuals of reproductive age (1). Some reproductive healthcare systems include screening for preventing risk factors, managing medical conditions, providing immunizations, supplementing iron and folic acid, modifying lifestyles and behaviors, and treating psychosocial problems (2). For these reasons, preconception care (PCC) is pivotal for improving pregnant women's birth outcomes and family health (3).

The leading cause of maternal fatalities worldwide is hemorrhage (27%), followed by preexisting diseases (15%), hypertension (14%), sepsis (11%), abortion (8%), and other indirect causes (7%) (4). Approximately 4 million children die in the first month of life, accounting for 40% of all deaths under the age of 5 years, with nearly all (98%) occurring in developing nations (5). During the period from 2018 to 2020, the death rate for children aged under 5 years was 42.5 per 1,000 live births (6). Each year, 527,000 pregnant women die from pregnancy-related problems in sub-Saharan Africa (7). Providing preconception care for reproductive-age couples is very important for improving the health of women, newborns, and children (8).

Although the fact that the Ethiopian Ministry of Health has launched new preconception care clinical guidelines (2) for improving reproductive health for women and couples, reducing maternal mortality and improving childbirth outcomes have not been achieved as sustainable goals (9). These new guidelines state that preconception care (assessing poor obstetric outcomes, such as early neonatal death, frequent abortion, stillbirth and birth defects, gestational diabetes mellitus, preterm birth, and small for gestational age babies) should be provided by knowledgeable and skilled clinicians before conception (10). For these reasons, preconception care has become a standard procedure and can be applied to every health facility (11, 12). Setting up a preconception care platform and implementing necessary packages at health facilities can reduce the risk of maternal, newborn, and child mortality and stillbirth (13, 14). However, there are still challenges in reducing maternal and childbirth mortality (15). Summarized evidence is needed to address these challenges to improve the quality and efficiency of healthcare service provision within the healthcare delivery system.

Even if some clinical guidelines are incorporated into maternal and child healthcare systems, maternal and child mortality are not significantly lower in Ethiopia (16). According to a systematic literature review of Ethiopian countries, the maternal mortality rate was 267 deaths for every 100,000 live births, the neonatal mortality rate was 29%, and the under-five mortality rate was 67 deaths for every 1,000 live births (17), 77% of which could be avoided by providing comprehensive healthcare services such as preconception care (18, 19). However, maternal and neonatal mortality reduction has not reached the intended level at the national level (20, 21). The findings showed that the prevalence of knowledge of preconception care among Ethiopian women was 30.95% (8). This finding showed that women's practice of preconception care was significantly low.

According to some primary studies, the practice of healthcare providers is affected by a lack of healthcare providers' awareness, inaccessible new guidelines, poor infrastructure for preconception care, and a lack of training, specifically on how to manage reproductive health problems in individuals who have poor obstetric care (22–25). Moreover, preventive measures at the national level are heavily dependent on integrating preconception care into the regular practices of healthcare providers (HCPs) to prevent this negative obstetric outcome (26). Although some primary studies have been conducted on HCPs' knowledge and associated factors, the studies on total healthcare providers in Ethiopia are inconsistent (27–30), which implies that there is no comprehensive review of the available evidence. Therefore, the purpose of this study was to evaluate healthcare providers' practice of preconception care and its associated factors in Ethiopian public health institutions.

## Methods

The current study examined healthcare professionals' practice of preconception care and related aspects while they were working in Ethiopian public health institutions. Letters, reviews, and commentaries were excluded from the study. The systematic review and meta-analysis were conducted in accordance with the Preferred Reporting Items for Systematic Reviews and Meta-Analyses (PRISMA) guidelines (31).

### Search strategy

#### Search strategy and review process

The authors conducted a comprehensive search using electronic databases (PubMed/Medline, Semantic Scholar, African Journal Online, and Embase) from 1 January 2023 to 2 March 2023. To ensure that no primary studies were overlooked, a confirmatory Google Scholar search was conducted. We looked through the digital holdings of the Ethiopian University of Science and Technology to find gray literature. The search strategies that were employed. Subsequently, a systematic review and meta-analysis were performed (Supplementary Data Sheet 1).

The search was restricted to research involving healthcare providers who worked in Ethiopia's public health facilities and who had a solid understanding of preconception care and related factors. Any differences were settled by consensus-building and discussion based on predetermined standards or by all the investigators in the event that a consensus could not be reached.

### Inclusion and exclusion criteria

#### Population

The study participants were healthcare providers (including nurses, public health officers, midwives, general practitioners, residents, and obstetric gynecologists) who work in the cancer screening ward and room. The modified “population, exposure, comparison, outcome, timeframe” (PECOT) framework (see Table 1) was used to construct the qualifying parameters for this review.

**Table 1:** Framework for determining the eligibility of studies (PECOT).

Criteria	Description
Population	The study participants were healthcare providers (including nurses, public health officers, midwives, general practitioners, residents, and obstetric gynecologists) who work in the cancer screening ward and room
Expose	Determinants of practice of PCC (sociodemographic data such as age, educational status, and the attitude and practice of healthcare) healthcare providers working at hospital health facilities, identifying poor obstetric health outcomes, healthcare providers reading PCC guidelines, being trained about HIV counseling and testing, and having access to a library nearly health facility and trained healthcare providers on PCC
Comparison	The reported reference groups for each determinant factor in each respective study such as practice of PCC among healthcare providers who have attended a degree and above vs. below a degree and above educational status, healthcare providers working at hospital health facilities vs. working at health centers, healthcare providers reading PCC guidelines vs. no more read PCC guidelines, healthcare providers who trained in HIV counseling and testing vs. untrained healthcare providers, and healthcare providers who had access to a library nearly health facility vs. their counterparts.
Outcome	Level of practice of PCC among healthcare providers
Type of study	Any observational type of study

This study included original research papers that described healthcare providers' level of practice of preconception care and related parameters while they worked in public health facilities in Ethiopia. Every publication released up until March 2023 G.C. was considered. Articles written only in English, whether published or unpublished, were also considered for inclusion. However, studies that did not explicitly state the level of knowledge of healthcare professionals with respect to preconception care and did not include the full texts of the studies were excluded. Moreover, editorial reports, letters, reviews, or commentary articles were excluded from this study.

#### Outcome measurements

The outcome variable of the study was the level of preconception care practice, and the second goal of the review was to identify the variables influencing Ethiopian healthcare providers' preconception care practice.

In most primary studies, the level of healthcare providers' knowledge of preconception care was assessed using similar numbers (15–20) of questions. A score greater than the mean score was used to indicate a good level of practice of preconception care (Table 2).

**Table 2:** Primary studies that used different methods to measure the prevalence of practice of preconception care among healthcare providers, in Ethiopia, 2023.

Articles	Measure the level of practice in preconception care
Mahlet MB, et al.	By 18 questions, answered ≥9 (good knowledge)
Andarg AK, et al.	By 20 questions, good practice (score 10–20)
Hawi A, et al.	From 15 practice-related questions. Considered to have good knowledge if scored 50th percentile
Berhane TA, et al.	Respondents who scored greater or equal to the mean (19.4%) on preconception care practice questions were categorized as having good PCC knowledge
Teketay DB, et al.	Respondents who correctly respond to 60% or more of the knowledge questions were categorized as having g ood practice
Seboka AS, et al.	Respondents who scored greater than or equal to the 50th percentile of the aggregated practice score were categorized as having good PCC knowledge
Eyerus N, et al.	From 20 knowledge questions, considered to have good practice if scored 10–20
Wolela AS, et al.	From 16 questions, considered to have good practice if answered correctly ≥9

#### Quality assessment

The modified Newcastle–Ottawa scale (NOS) was used to evaluate the quality of cross-sectional research, and the originality of each study was evaluated (32). Three primary components make up the assessment instrument. The tool's first section, which is rated on a scale of 1 to 5, evaluates the methodological quality of each study (including the sampling process, sample size, response rate, and determination of the risk factor or exposure). The comparability of the studies was evaluated in the second section of the tool, with the potential for two additional points to be awarded. The final part of the instrument rates the results and statistical analyses of the main study with a potential for three stars. The studies included in this systematic review and meta-analysis ranged from medium (6 out of 10) to high quality. Disagreements between reviewers during quality assessment were addressed through discussion.

#### Data processing and analysis

Standardized data were extracted using the standard Microsoft Excel format by two authors (AZ and YF). The following data were extracted from the included studies: name of authors, publication year, region, study area, sample size, study population, sampling technique, design, good preconception care, and risk factors. Three authors (YG, GA, and WT) revised the extracted data and discussed the data among the data extractors. The outcome data from the accepted research were combined and then exported into STATA version 11 software for analysis. The authors employed the random-effects model. The pooled prevalence of the outcome variable was reported with a 95% confidence interval. The *I*^2^ test was used to examine heterogeneity. We employed the subjective funnel plot observation method and Egger's test to evaluate publication bias. In terms of statistics, publication bias was identified at *p* < 0.05, and we also explored sources of heterogeneity through subgroup analysis by sampling technique and found heterogeneity in the sampling technique.

## Results

**Figure 1:**
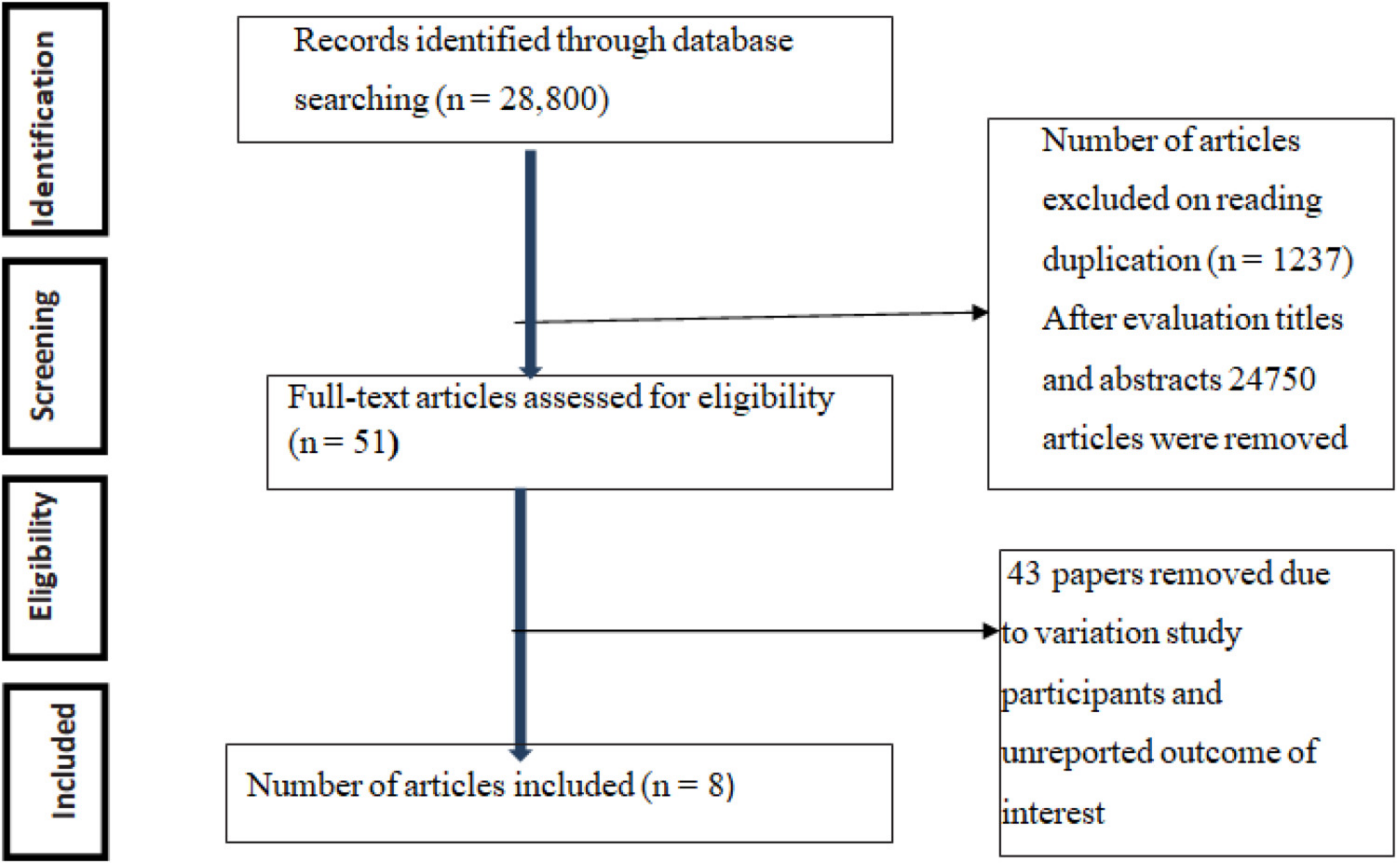
Flowchart showing records considered for systematic meta-analysis in Ethiopia.

A total of 28,800 articles concerning the practice of preconception care and/or associated factors among healthcare providers working in public health institutions in Ethiopia were assessed. Among the total retrieved papers, 1,237 were eliminated due to redundancy, and 24,750 articles were removed after being evaluated based on their titles and abstracts. After assessing the qualifying requirements of 51 papers, 43 papers were excluded due to variations in study participants and an unreported outcome of interest. Finally, eight papers were included in the meta-analysis (Figure 1).

### Characteristics of the included studies

All included studies used a facility-based cross-sectional study design to estimate the practice of preconception care. All studies were performed from 2018 to 2022. Five of the studies used simple sampling techniques (28–30, 33, 34), and three used multistage sampling techniques (3, 27, 35). From an estimated 3,848 healthcare providers, a total of 3,768 participants were involved, with an estimated sample size ranging from 156 (33) to 664 (35). The included studies reported that practice of preconception care ranged from 31.0% (3) to 97.1% (33). All the studies were performed in six regions (Amhara; Oromia; Addis Ababa; Southern Nations, Nationalities, and Peoples; Harreri; and Hawassa). Three of the studies included in this review were conducted in the Amhara region (27, 30, 35) (Table 3).

**Table 3:** Summary of the nine observational studies included in the meta-analysis assessing healthcare providers’ preconception care in Ethiopia, 2023.

First author	Region	Study area	Study year	Sampling technique	Sample size	Study percipients	Prevalence (%)	Overall quality
Hawi A (28)	Oromia	West Shoa Zone	2022	SRS	362	359	48.2	High
Mahlet MB (27)	Amhara	Awi Zone	2020	MSR	660	660	52	High
Andarg AK (3)	Hawassa	Hawassa	2018	MSR	634	634	31	High
Seboka AS (34)	Harari	Harer	2021	SRS	415	410	60.2	High
Teketay DB (30)	Amhara	Wollo Zone	2020	SRS	536	536	50.9	High
Berhane TA (29)	Benishangul-Gumuz	Assosa Zone	2022	SRS	421	416	66.1	High
Wolela AS (33)	Addis ababa	Tikur Anbessa	2019	SRS	156	130	69.2	Medium
Eyerus N (35)	Amhara	South Gondar Zone	2021	MSR	664	623	43.5	High

SRS, systematic random sampling, MSR, multistage random sampling.

### Preconception care knowledge

**Figure 2:**
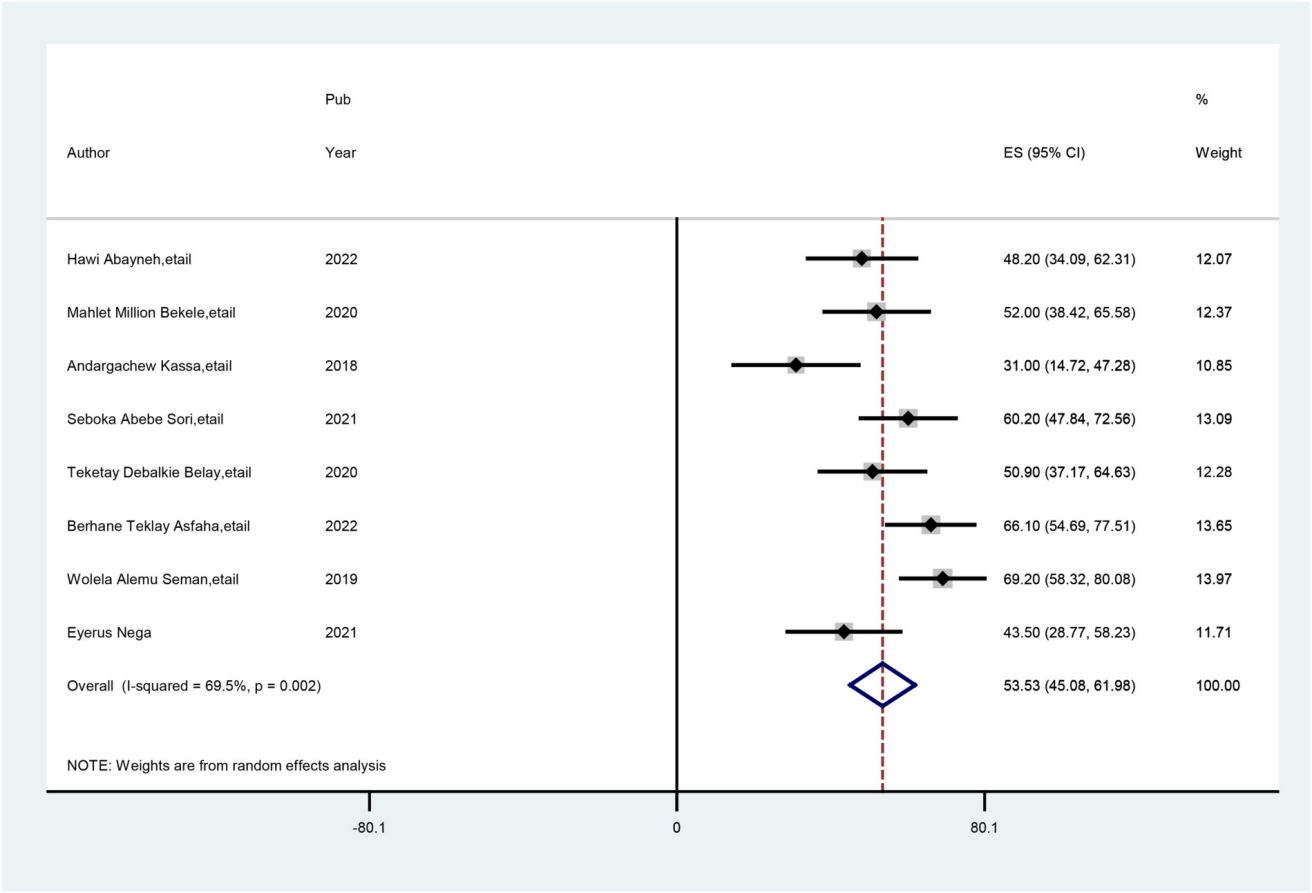
Forest plot of the pooled practice of preconception care among healthcare providers in Ethiopia.

The overall level of pooled preconception care practice among healthcare providers in Ethiopia was 53.5%. Substantial heterogeneity across the included studies was presented (*I*^2^ = 69.5%; *p* < 0.002) in the estimation of the prevalence of preconception care practice among healthcare providers. For this reason, a random-effects model was employed to determine the prevalence of preconception care practice among healthcare providers (Figure 2).

### Subgroup analysis

**Figure 3:**
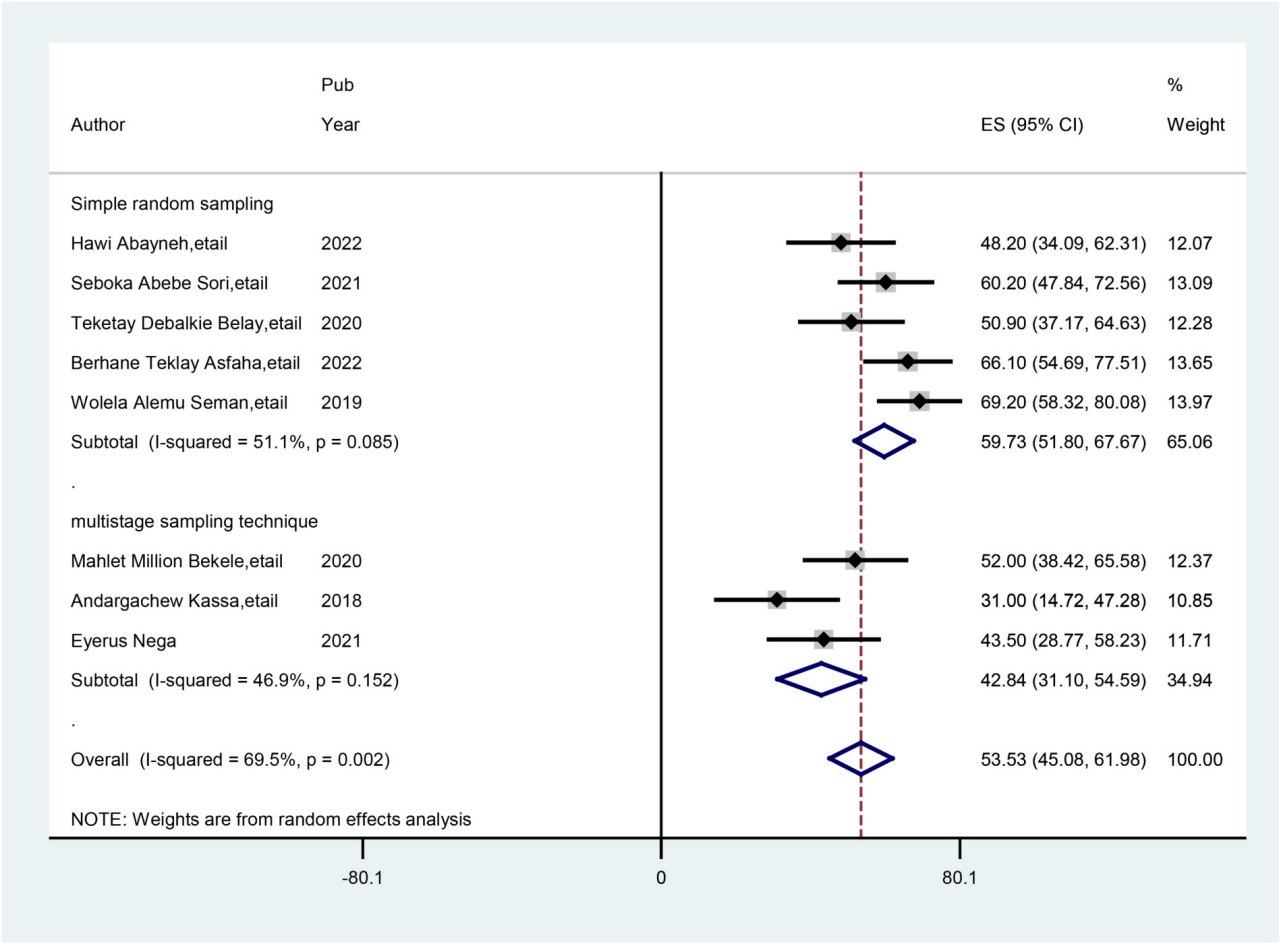
Subgroup analysis (by sampling technique) of studies included in the meta-analysis on practice of preconception care among healthcare providers in Ethiopia.

Subgroup analysis was carried out basis of the sampling technique used in the primary studies. Accordingly, the highest prevalence of practice of preconception care was observed with simple random sampling techniques at 59.73% (95% CI: 51.80, 67.67), and the lowest prevalence of practice of preconception care was observed with multistage sampling techniques, at 42.84% (95% CI: 31.10, 54.59) (Figure 3).

#### Publication bias

**Figure 4:**
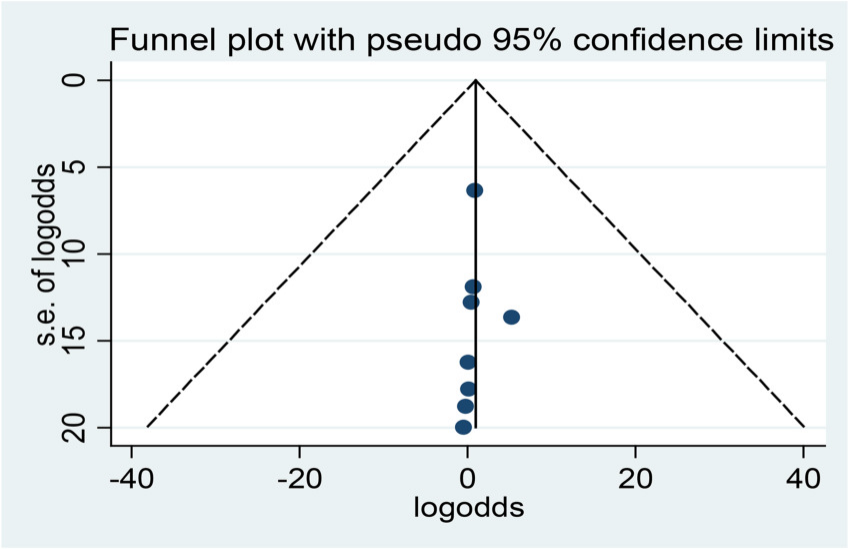
Graphical representation of publication bias using funnel plots of all included studies.

Egger's regression test and a visual assessment of the asymmetry in funnel plots were used to determine whether publication bias existed. As a result, there was no publication bias from the findings. Egger's test ruled out the absence of publication bias (*p* = 0.865). The funnel plots also revealed a symmetrical distribution of studies upon visual observation (Figure 4).

### Factors associated with practice of preconception care

This comprehensive review and meta-analysis revealed that practice of preconception care among Ethiopian healthcare providers was strongly associated with educational status, healthcare providers who worked at hospital facilities, those who read preconception care guidelines, those trained in HIV counseling and testing, those receiving preconception care, and those with access to a library near a health facility.

**Figure 5:**
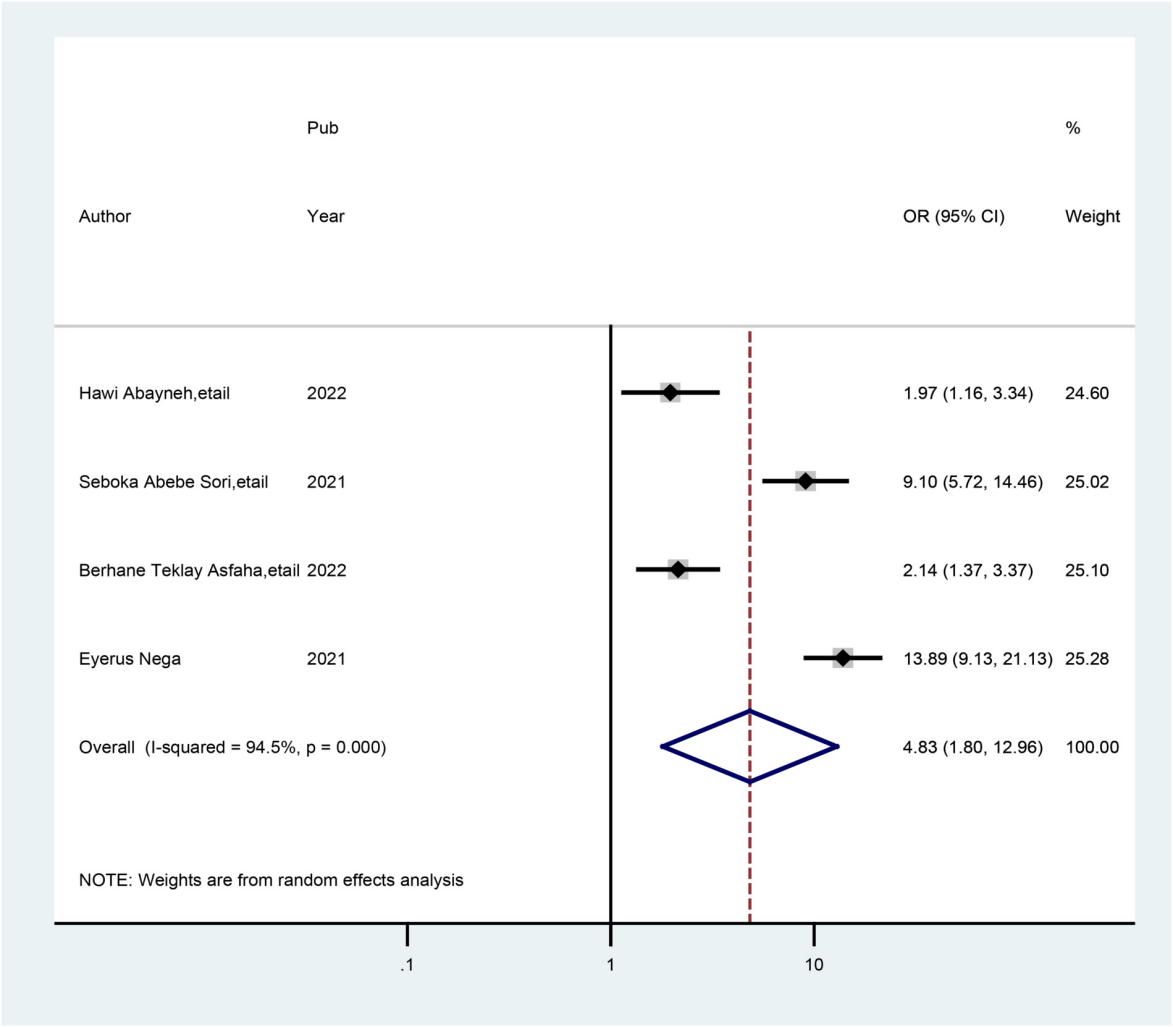
Forest plot showing the pooled odds ratio of the association between healthcare providers who have a degree educational status and good practice of preconception care.

According to this study, four studies indicated that healthcare providers who had a degree and above education had a significant association with good practice of preconception care. The odds of having good practice of preconception care were 4.83 times greater (AOR = 4.83; 95% CI: 1.80, 12.96) among healthcare providers who had a degree and above holders' education status in preconception care than among those who had below-degree holders' education status. The studies included in this meta-analysis were marked by heterogeneity (*I*^2^ = 97.0%, *p* = 0.000). As a result, a random-effects model was performed (Figure 5).

**Figure 6:**
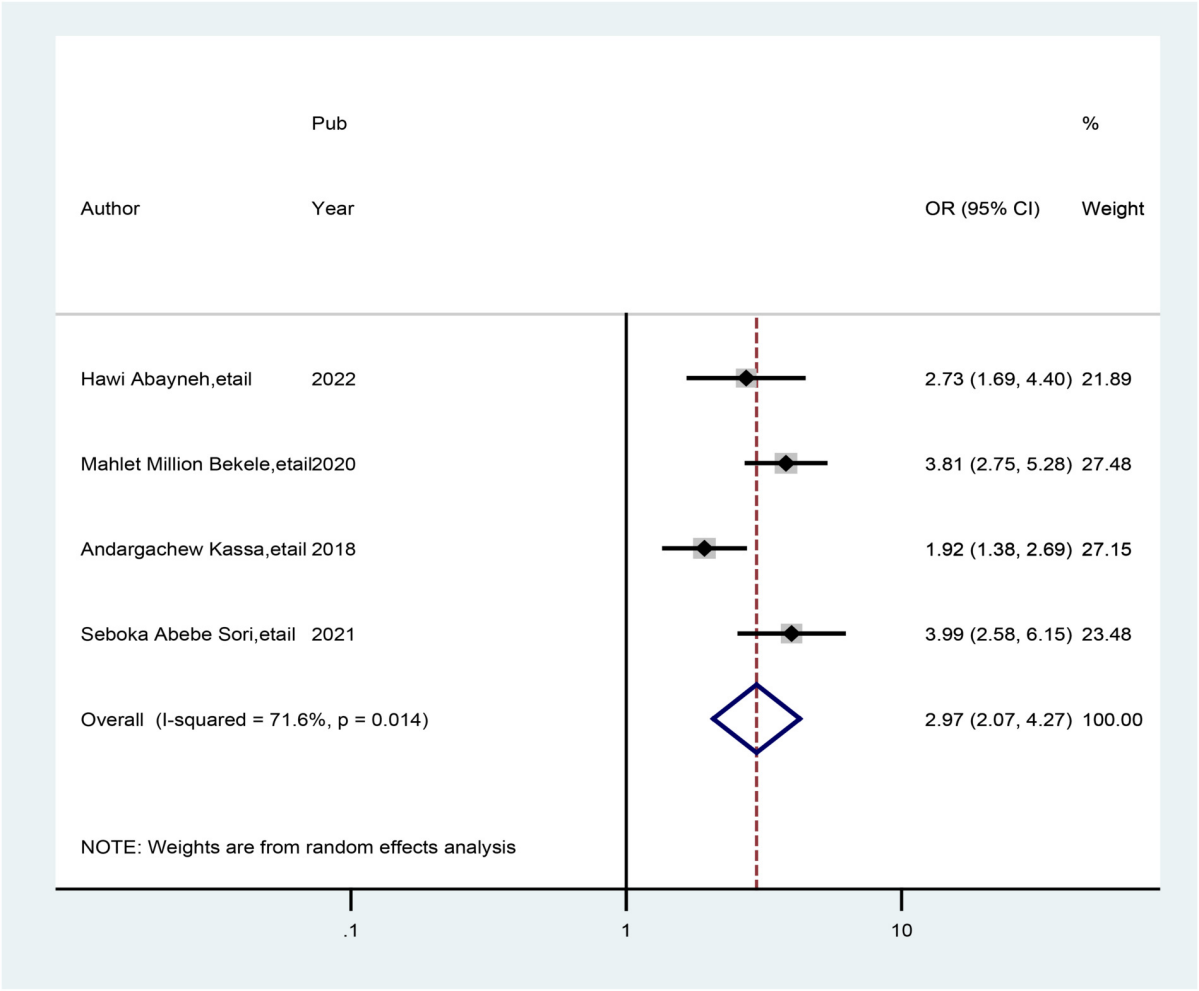
Forest plot showing the pooled odds ratio of the association between healthcare providers.

Four studies indicated that healthcare providers who worked at hospital health facilities had a significant association with good practice of preconception care. The odds of having practice of preconception care were 2.97 times (AOR = 2.97; 95% CI: 2.07, 4.27) greater among healthcare providers who worked at hospital health facilities than among those who worked at health centers. In this meta-analysis, the included studies exhibited heterogeneity (*I*^2^ = 68.1%, *p* = 0.024) (Figure 6).

**Figure 7:**
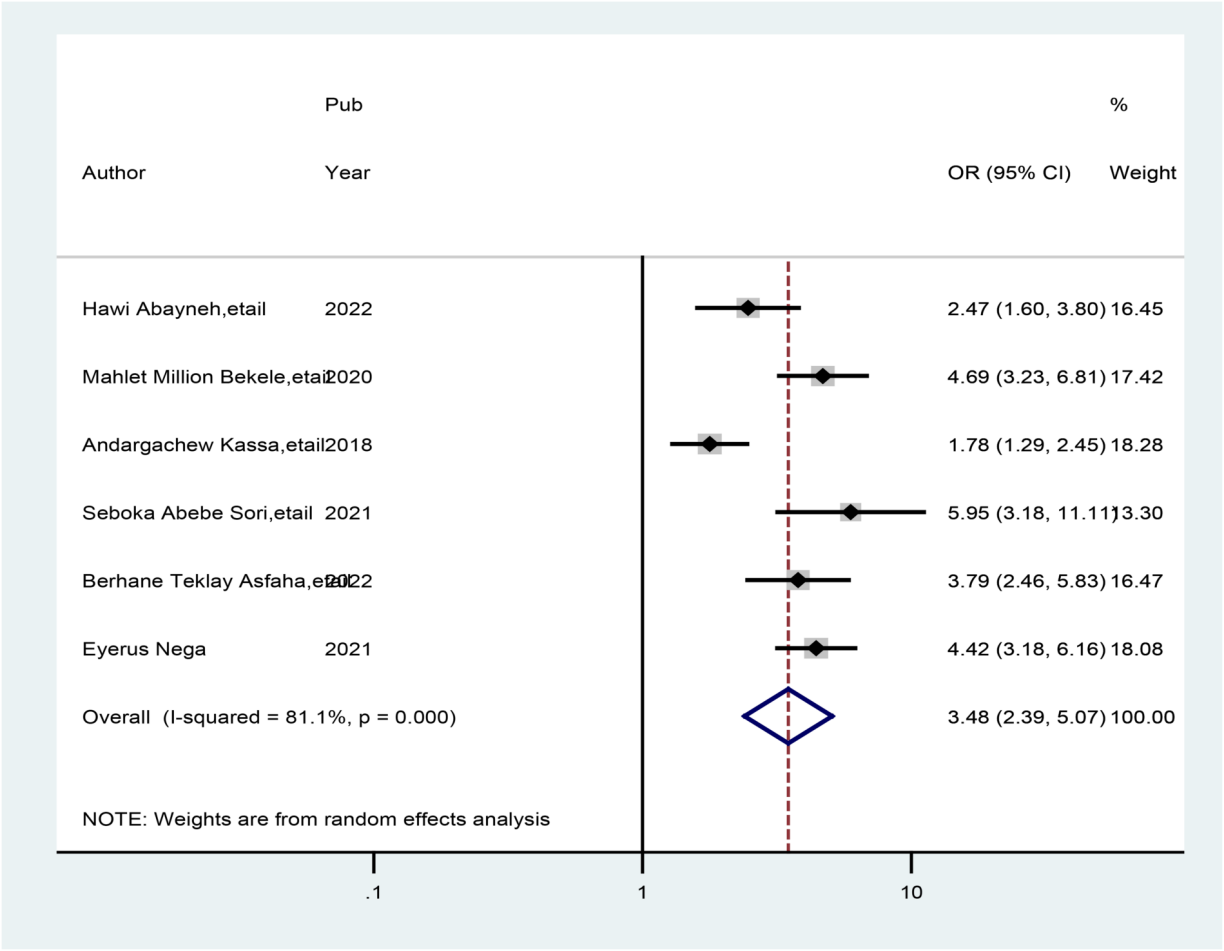
Forest plot showing the pooled odds ratio of the association between healthcare providers who read preconception care guidelines and good practice of preconception care.

Six studies indicated that healthcare providers who had ever read PCC guidelines had a significant association with good practice of preconception care. Hence, healthcare providers who read preconception care guidelines were 3.49 times (AOR = 3.49; 95% CI: 2.39, 5.07) more likely to have good practice of preconception care than those who did not read the preconception care guidelines. A random-effects model was used in this meta-analysis because the included studies were characterized by the existence of heterogeneity (*I*^2^ = 77.9%, *p* = 0. 000) (Figure 7).

**Figure 8:**
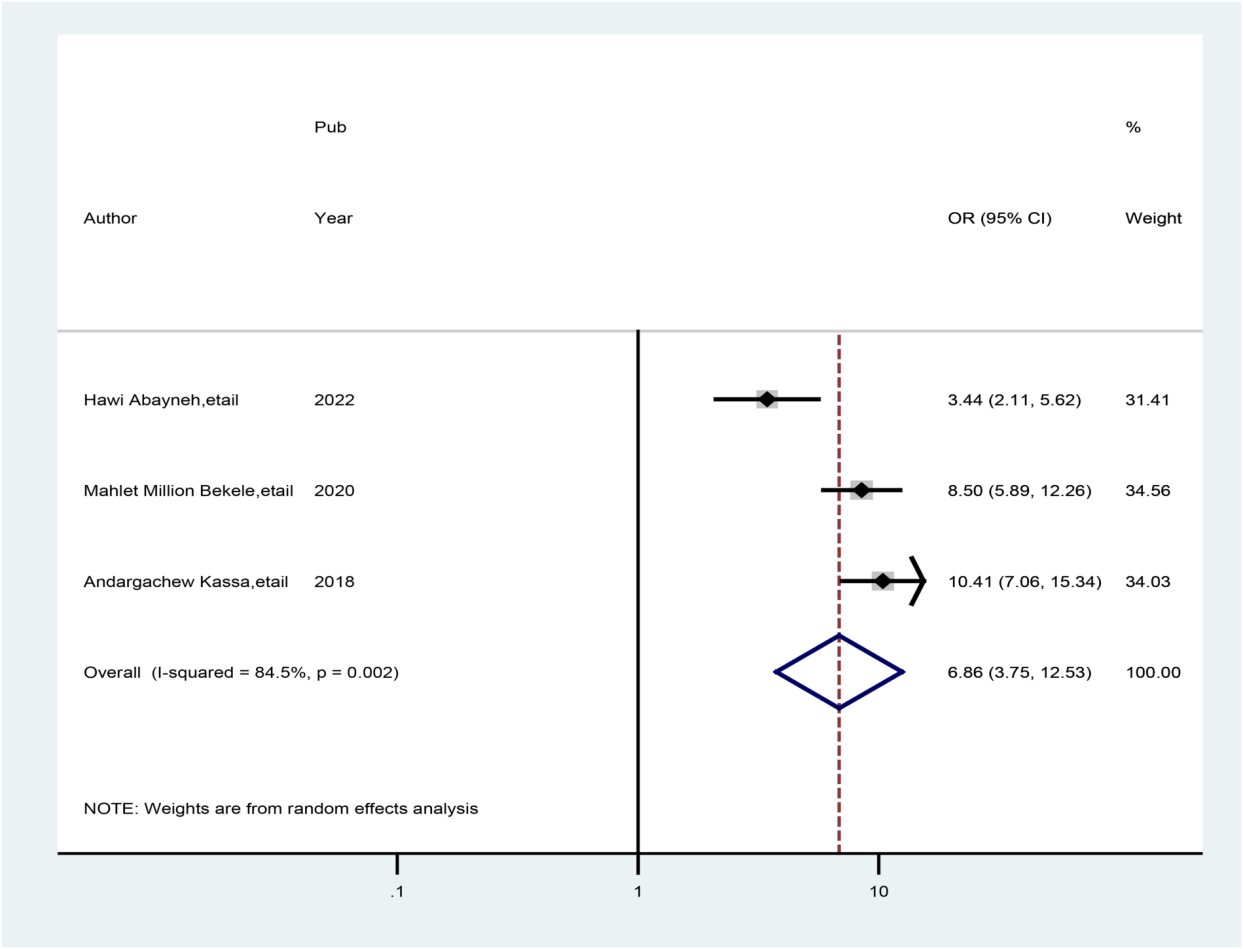
Forest plot showing the pooled odds ratio of the association between healthcare providers who read preconception care guidelines and good practice of preconception care.

Three studies also showed that healthcare providers being trained in HIV counseling and testing were associated with good practice of preconception care. The odds of good practice of preconception care were 6.86 times greater (AOR = 6.86; 95% CI: 3.75, 12.53) among healthcare providers who were trained in HIV counseling and testing than among their counterparts. A random-effects model was used in this meta-analysis due to the presence of heterogeneity (*I*^2^ = 86.6%, *p* = 0.001) (Figure 8).

**Figure 9:**
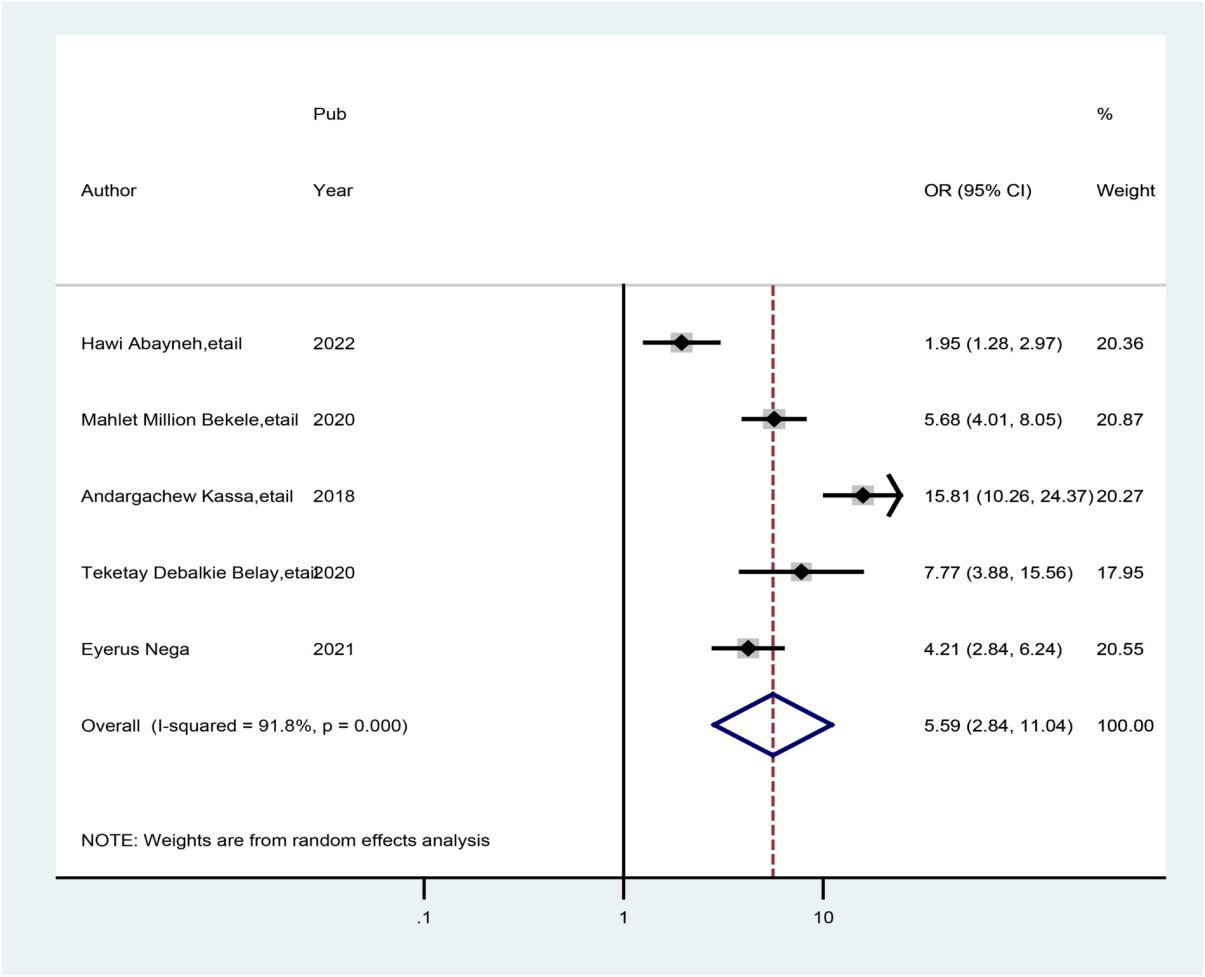
Forest plot showing the pooled odds ratio of the association between the availability of libraries for health stakeholders and good practice of preconception care.

Moreover, five studies also showed that the availability of libraries at health facilities was associated with preconception care and good practice. The odds of having preconception care practice were approximately 5.60 times greater (AOR = 5.59; 95% CI: 2.84, 11.04) among healthcare providers who had libraries than among their counterparts. We used a random-effects model to reduce the presence of high heterogeneity (*I*^2^ = 93.1%, *p* = 0.000) (Figure 9).

**Figure 10:**
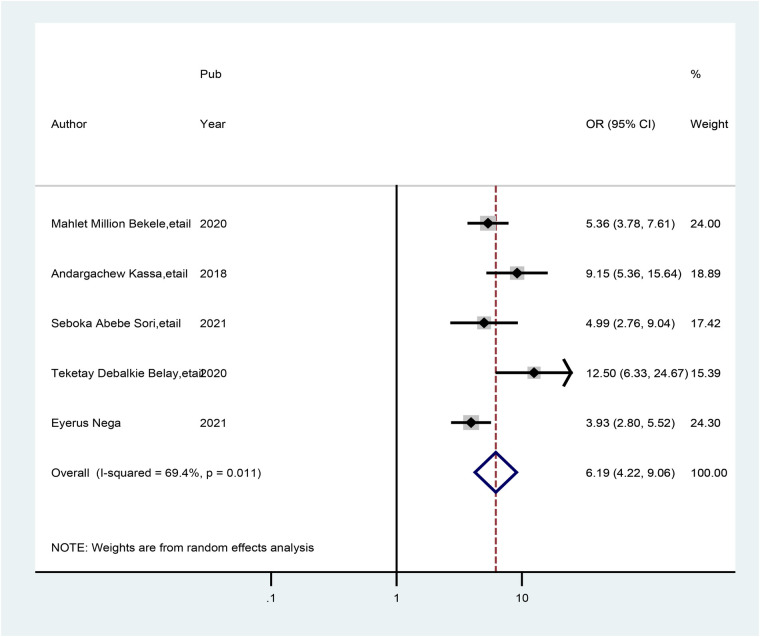
Forest plot showing the pooled odds ratio of the association between healthcare providers who received preconception care training and those with good practice of preconception care.

Finally, five studies revealed a correlation between healthcare providers who were trained in preconception care and those who were not trained in preconception care. Hence, this association showed that healthcare providers who were trained in preconception care had more practice than their counterparts. The odds of practice of preconception care were approximately 6.20 times greater (AOR = 6.19; 95% CI: 4.23, 9.06) among those trained in preconception care healthcare providers than among those who were not trained in preconception care. We also performed a meta-analysis and employed a random-effects model to prevent high levels of heterogeneity across the included studies (*I*^2^ = 82.8%, *p* = 0.000) (Figure 10).

## Discussion

The purpose of this systematic review and meta-analysis was to determine the level of good practice and its associated factors in preconception care among healthcare providers. The evidence was summarized based on the good practice and determinants of preconception care among healthcare providers who worked at health facilities in Ethiopia. Accordingly, healthcare providers have attempted to gather sufficient practice on preconception care, which plays a crucial role in good birth outcomes. This meta-analysis revealed that 53.53% (95% CI: 45.09, 61.98) of healthcare providers had a good level of practice of preconception care. This finding implies that the need for preconception care awareness and the implementation of the Ethiopian Ministry of Health's preconception care guidelines for all healthcare providers, as well as healthcare, will help promote preconception care at the healthcare system level. Furthermore, this finding infers the need to make plans once practice gaps are identified. Strengthening preconception care by focusing on determinants of good practice among healthcare providers is one of the most important components for improving the quality of preconception care (29). Healthcare providers' levels of practice of preconception care and associated factors are needed to achieve good long-term maternal and child health outcomes.

Although preconception care services are available for all healthcare system providers and different primary studies were available, no comparable meta-analysis has been carried out on this topic at the national level. This finding is in line with those of studies conducted in Nigeria (58.3%) (36) and Qatar (53.7%) (37). These similarities might be due to the similarities in educational status, policies and guidelines used, and healthcare approaches used in developing countries. This result suggested that preconception care strategies/activities for raising awareness were insufficient, but further studies are needed to increase healthcare practitioners' practice of these strategies/activities. To effectively implement preconception care at every health facility, it is necessary to involve both governmental and non-governmental stakeholders in providing preconception care.

However, this figure is lower than that of survey studies carried out in China (90%) (38), Canada (70%) (39), and the USA (76%) (40). The possible reasons might be the low socioeconomic status, differences in the infrastructure of the health sector, lack of health promotion of preconception care reported in the media coverage for preconception care across the county, lack of preconception clinics at the health institution level, and low commitment of healthcare workers due to the high case flow of clients in Ethiopia, which could be contributing factors to the low level of practice reported in this study.

This level of good practice of healthcare providers (53.53%) was also higher than that reported in a study conducted in sub-Saharan Africa (24.5%) (41) and studies among women in Ethiopia (35.7%) (42). These discrepancies could be due to differences in the educational status of the study participants. This funding implies that our study participants were healthcare providers, which may increase their practices of preconception care and its implementation by the national healthcare system. Therefore, multiple stakeholders need to be involved to further improve the practice of healthcare providers about preconception care.

This meta-analysis revealed that healthcare providers who had a degree and above were nearly five times more likely to have good practice of preconception care than healthcare providers who had a below-degree education (AOR = 4.83; 95% CI: 1.80, 12.96). Similar findings were comparable to the outcomes reported in studies in Sudan (42), Africa (43), and America (44). These results might be explained by the fact that as the educational level of healthcare providers increases, so do professionals' positive attitudes toward preconception care. More highly educated (degree and above) healthcare providers might therefore be more inclined to offer preconception care. Additionally, more educated health professionals can have easier access to or a stronger desire to seek out sources of practice pertaining to their well-being and have better preconception care management strategies for their reproductive health system.

Similarly, healthcare providers who worked at hospital health facilities had almost three times more practice of preconception care than those who worked at health centers (AOR = 2.97; 95% CI: 2.07, 4.27). The findings presented here were in parallel with those of studies conducted in the USA (45) and England (46). This study suggested that providing preconception care at hospital health facilities may involve more experienced physicians and more accessible medications than preconception care at health center facilities. Healthcare providers know that healthcare providers who work at comprehensive hospital levels may have much work experience and increased levels of good practice of preconception care.

The current meta-analysis also indicated that healthcare providers who had ever read preconception care guidelines were 3.49 times more likely to have good practice of preconception care than their counterparts (AOR = 3.49; 95% CI: 2.39, 5.07). This finding is supported by a study performed in Australia (47). Moreover, this study demonstrated that healthcare providers from health institutions that had plans for preconception care had more practice of preconception care than their counterparts. This is supported by the fact that healthcare providers should read preconception care guidelines (48). Policies, guidelines, recommendations, and services related to preconception care are crucial for identifying, controlling, and treating risk factors that have an impact on obstetric outcomes (49, 50). Therefore, healthcare providers who have ever read such guidelines may have good practice and motivation to implement preconception care.

The current meta-analysis also revealed that practice scores increase with training on HIV testing and counseling to provide preconception care. As a result, healthcare providers who were trained in HIV testing presented with good practice scores on preconception care (AOR = 6.86; 95% CI: 3.75, 12.53) and 6.19 times (AOR = 6.19; 95% CI: 4.23, 9.06) more good practice of preconception care among trained healthcare providers on related preconception than their counterparts. This might be due to the impacts of the attention given to the prevention of HIV/AIDS transmission and improving birth outcomes, as government agenda implementation enables health providers' practice to improve preconception care (27, 43). As a result, healthcare providers may gain a good level of practice in overall gynecological and obstetrical healthcare services.

Moreover, the availability of a library at a health facility was approximately 5.60 times greater when preconception care was provided than when healthcare providers did not have a library at a public health facility (AOR = 5.59; 95% CI: 2.84, 11.04). This association might be due to the library's access to preconception care information. These associations are supported by a systematic review among reproductive-age women and couples, which showed that those available in a library at a health facility had a broader understanding of complications associated with being unable to receive preconception care than their counterparts (43, 51). A good level of practice of preconception care services should be improved by reading preconception care guidelines at the library or by using smartphones. For these reasons, the Ethiopian Ministry of Health should design libraries and implement important preconception care guidelines at nearby health facilities.

Despite its considerable value, this systematic review and meta-analysis have several limitations. First, the result variable may be influenced by other confounding variables, such as misunderstandings, practice, and accessibility of the service, as all of the research included in this review was cross-sectional. Second, small sample sizes in some of the primary studies that were part of this systematic review and meta-analysis could have an impact on the true level of practice at the national level. Third, because of the various categories, a few significant variables were overlooked. Lastly, only five regions and one administrative town in Ethiopia were included in this study's review because of the nation's small number of primary studies.

### Relevance to research

The findings of this study will serve as a roadmap for future research to provide an in-depth understanding of preconception care and its variables in Ethiopia. Future policymakers should pay special attention to lowering maternal and child morbidity and death associated with the inability to provide preconception care. Research should also concentrate on advancing the understanding of the sociodemographic and other factors associated with poor preconception care to tailor health promotion initiatives for the most vulnerable segments of the reproductive-age population and future generations and to provide individualized coaching and information to improve nutrition and lifestyle during the preconception period.

## Conclusions and recommendations

Nearly half of healthcare providers do not have a good level of practice of preconception care in Ethiopia. For this low level of practice of preconception care, healthcare providers' low educational status, lack of training in preconception care, HIV testing, absence of guidelines, libraries, and lack of internet access at nearby health facilities were significant factors. Hence, stakeholders and policymakers should work on strategies and policies to improve healthcare providers' level of practice of preconception care by accessing internet services and mini-libraries near health facilities. In addition, we wish to recommend to the Ethiopian Ministry of Health and Health Bureaus that they organize and provide continuous refreshment training on preconception care, HIV counseling and testing and make the available guidelines for diploma-level health workers. Moreover, to obtain more comprehensive evidence, we recommend further scoping reviews.
